# 1617. Assessment of Bloodstream Infections in Patients with Intrapartum Fever

**DOI:** 10.1093/ofid/ofad500.1452

**Published:** 2023-11-27

**Authors:** Barbara Kamel, Thien-Ly Doan, Vidal Luchana, Michael Oppenheim, Henry Donaghy

**Affiliations:** Long Island Jewish Medical Center, New Hyde Park, New York; Long Island Jewish Medical Center, New Hyde Park, New York; Long Island Jewish Medical Center, New Hyde Park, New York; Northwell Health, Lake Success, New York; Northwell Health, Lake Success, New York

## Abstract

**Background:**

The American College of Obstetricians and Gynecologists (ACOG) offers guidance on the management of intraamniotic infections, where the diagnosis of chorioamnionitis can be made based on clinical criteria (e.g., maternal leukocytosis, purulent cervical drainage, or fetal tachycardia), positive amniotic fluid analysis (gram stain, glucose level, cultures), or placental pathology. No recommendations for blood cultures are made. The study purpose is to evaluate the utility of blood cultures in the management of intrapartum fever and to analyze the microbiological data.

**Methods:**

This is a retrospective, descriptive study that analyzes patients with blood cultures drawn within 5 days of delivery from 1/2019 to 12/2022. Procurement contaminants were excluded from the analysis. Chart reviews were conducted on the positive cultures. Data collected included age of mother, gestational age, method of delivery, presence of fetal tachycardia, cultures sent prior to admission, maternal Group B *Streptococcus* (GBS) status, culture results including organism (blood, placenta cultures), and antimicrobials administered. Descriptive statistics were utilized.

**Results:**

Of the 70,540 deliveries in the study period, there were a total of 507 patients had blood cultures drawn, of which 25 were positive. Three patients were excluded because they were deemed to be procurement contaminants (e.g., 2 patients with coagulase-negative *Staphylococcus* species and 1 patient with *Micrococcus* species). Analysis of the bloodstream infection were performed on 22 patients, thus the rate of bacteremia was 4.3% in our cohort. The mean age was 33.3 ± 4.4 years with an average gestational age at birth of 32.3 ± 6.5 weeks. Of the 22 patients, 19 (86.4%) received a cesarean section and 4 (18.2%) were positive for GBS. The most common pathogen present were *E. coli (*45.5%) and *Prevotella bivia* (13.6%). The most common antimicrobials given were ertapenem (63.6%), followed by clindamycin + gentamicin (13.6%).

Organisms Isolated from Blood Cultures

**Figure:**
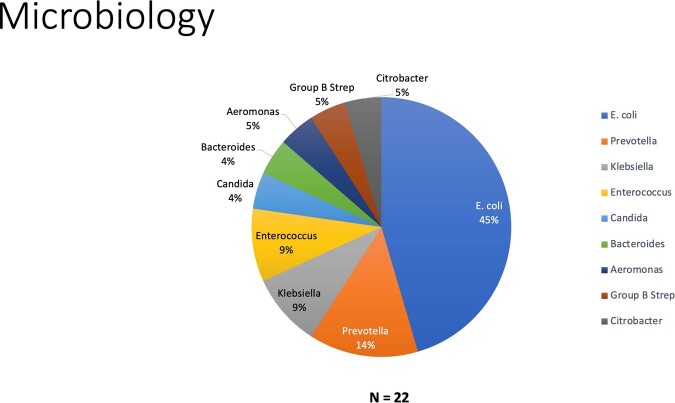

E. coli was the predominant organism isolated, followed by Prevotella. Interestingly, Candida was isolated in 1 patient where empiric ertapenem or gentamicin + clindamycin would not have been sufficient.

**Conclusion:**

Although uncommon, bacteremia can occur in patients with intrapartum fevers. Gram-negative and anaerobes were found to be most common organisms. Bloodstream infections may require a longer duration of treatment therefore blood cultures should be considered as part of the work-up of intrapartum fever.,

**Disclosures:**

**All Authors**: No reported disclosures

